# Dynamics of androgens in healthy and hospitalized newborn foals

**DOI:** 10.1111/jvim.15974

**Published:** 2020-12-05

**Authors:** Jacob M. Swink, Lindsey M. Rings, Hailey A. Snyder, Rachel C. McAuley, Teresa A. Burns, Katarzyna A. Dembek, William F. Gilsenan, Nimet Browne, Ramiro E. Toribio

**Affiliations:** ^1^ College of Veterinary Medicine, The Ohio State University Columbus Ohio USA; ^2^ Rood and Riddle Equine Hospital Lexington Kentucky USA; ^3^ College of Veterinary Medicine, North Carolina State University Raleigh North Carolina USA; ^4^ Hagyard Equine Medical Institute Lexington Kentucky USA

**Keywords:** androgens, equine neonate, foal, progestogens, sepsis, sex steroids, steroidogenesis

## Abstract

**Background:**

Information on steroids derived from the adrenal glands, gonads, or fetoplacental unit is minimal in newborn foals.

**Objective:**

To measure androgen concentrations in serum and determine their association with disease severity and outcome in hospitalized foals.

**Animals:**

Hospitalized (n = 145) and healthy (n = 80) foals.

**Methods:**

Prospective, multicenter, cross‐sectional study. Foals of ≤3 days of age from 3 hospitals and horse farms were classified as healthy and hospitalized (septic, sick nonseptic, neonatal maladjustment syndrome [NMS]) based on physical exam, medical history, and laboratory findings. Serum androgen and plasma ACTH concentrations were measured with immunoassays. Data were analyzed by nonparametric methods and univariate analysis.

**Results:**

Serum dehydroepiandrosterone (DHEA), androstenedione, testosterone, and dihydrotestosterone (DHT) concentrations were higher upon admission in hospitalized foals (*P* <  .05), were associated with nonsurvival, decreased to 4.9‐10.8%, 5.7‐31%, and 30.8‐62.8% admission values in healthy, SNS, and septic foals, respectively (*P* < .05), but remained unchanged or increased in nonsurviving foals. ACTH:androgen ratios were higher in septic and NMS foals (*P* < .05). Foals with decreased androgen clearance were more likely to die (odds ratio > 3; *P* < .05).

**Conclusions and Clinical Importance:**

Similar to glucocorticoids, mineralocorticoids, and progestagens, increased serum concentrations of androgens are associated with disease severity and adverse outcome in hospitalized newborn foals. In healthy foals, androgens decrease over time, however, remain elevated longer in septic and nonsurviving foals. Androgens could play a role in or reflect a response to disorders such as sepsis or NMS in newborn foals.

**Abbreviations**
CBG: cortisol binding globulin
CI: confidence interval
CIRCI: critical illness‐related corticosteroid insufficiency
DHEA: dehydroepiandrosterone
DHEA‐S: dehydroepiandrosterone sulfate
DHP: dihydroprogesterone
DHT: dihydrotestosterone
GABAA: gamma‐aminobutyric acid (GABA) type A
GABAAR: GABAA receptor
LC/MS: liquid chromatography mass spectrometry
NMDA: N‐methyl‐D‐aspartate
NMDAR: NMDA receptor
NMS: neonatal maladjustment syndrome
OR: odds ratio
RAI: relative adrenal insufficiency
SHBG: sex steroid binding globulin
SNS: sick nonseptic

## 1 INTRODUCTION

Sepsis remains the leading cause of death in newborn foals.^1^ Cortisol is the main steroid released by the adrenal gland and in the developing fetus is essential for tissue maturation and differentiation.^2^, ^3^ A number of critically ill foals have an insufficient cortisol response to stress, but adequate to abnormally high ACTH concentrations, a phenomenon known as relative adrenal insufficiency (RAI) or critical illness‐related corticosteroid insufficiency (CIRCI).^4^, ^5^ In addition to stimulating glucocorticoid and mineralocorticoid production, ACTH also promotes the secretion of steroid precursors and androgens from the adrenal cortex and developing gonads.^6^, ^7^

Pregnenolone and dehydroepiandrosterone (DHEA) are the main steroid precursors in the equine fetus and their source are the fetal gonads and adrenal glands.^8^ These steroids are metabolized by the equine placenta into progestogens, androgens, and estrogens, which are transferred to both fetal and maternal circulation,^9^ where they perform specific functions on fetal development and pregnancy maintenance.^8^, ^10^ Steroid concentrations in blood of newborn foals likely reflect fetal concentrations before parturition, which decrease rapidly after birth.^11^, ^12^, ^13^ Increased concentrations of glucocorticoids, mineralocorticoids, and progestogens in blood have been associated with various disorders of newborn foals.^11^, ^14^, ^15^, ^16^, ^17^, ^18^ However, little is known about the role and kinetics of androgens in healthy and sick equine neonates.

Cortisol, aldosterone, progestogens, and DHEA concentrations are elevated in sick foals and linked to disease severity,^11^, ^14^, ^15^, ^16^, ^17^, ^18^ and their response to exogenous ACTH stimulation is associated with outcome.^4^ Most studies of steroid concentrations in foals have been on single time measurements, precluding the assessment of steroid dynamics in relation to illness severity and outcome over time. Information on androgen concentrations in newborn foals is lacking, likely because they are seen as sex hormones rather than active steroids or intermediary to other endocrine factors, further underplaying their clinical measurement and relevance in healthy and sick foals.

Progestogens are neuroprotective and could play a role in the pathogenesis of equine neonatal maladjustment syndrome (NMS).^17^, ^19^ Progestogens such as allopregnanolone have sedative effects in healthy foals.^19^ Elevations of serum progestogens and androgens (DHEA and androstenedione) have been associated with NMS.^17^ Although most data on neurosteroids have been on progestogens, androgens also modulate neural receptors.^20^, ^21^

DHEA and androstenedione are the main androgen precursors found in circulation of pregnant mares and foals, whereas testosterone and dihydrotestosterone (DHT; 5α‐dihydrotestosterone) are considered the most active androgenic metabolites. They modulate GABA_A_ and N‐methyl‐D‐aspartate receptors (NMDAR).^20^, ^22^ Relevant to the equine neonate, DHEA and androstenedione concentrations have been measured in sick and healthy foals, but testosterone and DHT concentrations have not been assessed.^11^, ^15^

In this study, we aimed to investigate androgens in healthy and sick neonatal foals over time and to determine their association with severity of disease and outcome. The ACTH to androgen relationship (ACTH:androgen ratios) were also investigated. We hypothesized that septic foals will have higher serum androgen concentrations that will remain elevated longer than in sick nonseptic (SNS) and healthy foals. We also proposed that steroid elevation would be associated with likelihood of death.

## 2 MATERIALS AND METHODS

### 2.1 Animals

A total of 225 newborn foals ≤3 days of age of any breed and sex from the 2017‐2019 foaling seasons were included in this prospective study. Hospitalized foals (n = 145) were admitted to 3 equine referral hospitals (masked for review), whereas healthy foals (n = 80) were evaluated at the farm or hospitals.

Hospitalized foals with a positive blood culture or a sepsis score ≥12 were considered septic (n = 81),^23^ whereas foals who presented for illnesses other than sepsis (eg, retained meconium, orthopedic conditions), negative blood cultures, and sepsis scores ≤11 were classified as SNS (n = 64).

Foals ≤3 days of age with normal physical examination, CBC, serum biochemistry profile, serum immunoglobulin G >800 mg/dL, and sepsis scores <4 were classified as healthy. Foals were also classified into NMS (n = 63) or sick non‐NMS (n = 82) groups based on clinical diagnosis.^24^ Based on survival, hospitalized foals were categorized into survivors (n = 109) and nonsurvivors (n = 36). Survivors were foals discharged alive and nonsurvivors were foals that died or were euthanized because of a grave medical prognosis. Foals euthanized for nonmedical reasons (eg, finances) were not included in this study.

This study was approved by (masked for review) and adhered to the principles of humane treatment of animals in veterinary research, as stated by the American College of Veterinary Internal Medicine and the National Institutes of Health Guidelines.

### 2.2 Sampling

Blood samples from hospitalized foals were collected upon admission (0 hour) and at 24, 48, and 72 hours after admission. Blood from healthy foals were collected during their routine newborn foal examination at 18 to 30 hours of age (time 0) and 24, 48, and 72 hours later. Blood samples were collected into serum clot and EDTA‐aprotinin tubes, centrifuged at 2000*g* for 10 minutes, aliquoted and stored at −80°C until analysis.

### 2.3 Determination of hormone concentrations

Radioimmunoassays were used to measure serum concentrations of DHEA (MP Biomedicals, Solon, Ohio), testosterone (Tecan International, Morrisville, North Carolina), and androstenedione (Tecan International). Serum concentrations of DHT were measured using an ELISA (DRG International, Springfield Township, New Jersey). All assays measured total concentrations and had ≤6% cross‐reactivity to other androgens. These assays showed linearity for equine serum samples at dilutions up to 1:8 and had intra‐ and interassay coefficients of variation of <10%. Plasma ACTH concentrations were determined using a human‐specific immunochemiluminometric assay previously validated for equine samples (Immulite, Siemens, Los Angeles, California).^25^ Three of these assays have been previously validated in equids.^15^

### 2.4 Statistical analysis

Data were assessed for normality using the Shapiro‐Wilk test and found to be not normally distributed. Therefore, results are presented as medians, interquartile ranges, and 95% ranges. Comparisons among 3 groups (healthy vs SNS vs septic; healthy vs sick non‐NMS vs NMS) were performed using the Kruskal‐Wallis ANOVA with Dunn's post hoc analysis. Comparisons between 2 groups (colt vs filly; survivor vs nonsurvivor) were done with the Mann‐Whitney *U* test. Proportions were compared with the Chi‐square test. Comparison of hormone changes over time was carried out with the Friedman's test. Sick foals were categorized as having low, high, and within normal hormone concentrations by using 95% confidence interval (CI) values from healthy foals as cutoff points.

Based on previous studies showing that serum progestogen and androgen concentrations are high in healthy newborn foals, and decrease steadily a few days after birth,^11^, ^13^ we defined this reduction as steroid clearance (delta, Δ). Steroid Δ values (Δ_0‐24,_ Δ_24‐48,_ Δ_48‐72,_ Δ_0‐72_) were calculated by subtracting the later time from the earlier time concentration. Therefore, foals with decreases in serum androgen concentrations over time will have Δ values >0, whereas foals with increases or no changes in serum androgen concentrations over time will have Δ values ≤0. In addition, androgen percentage changes for each group at 24, 48, and 72 hours were calculated relative to time zero. ACTH:androgen ratios were calculated by dividing the plasma ACTH concentration by its matching androgen concentration at a given time point.

Univariate logistical regression and crude odds ratios (OR) for nonsurvival were determined. The referent was the high‐end of the healthy foal 95% CI. Statistical and graphical software used included IBM SPSS 26 (IBM Corporation, Armonk, New York), GraphPad Prism 8 (GraphPad Software, Inc, La Jolla, California), and SigmaPlot 14.0 (Systat Software, Inc, San Jose, California). Significance was set at *P* < .05.

## 3 RESULTS

### 3.1 Population

Of the hospitalized foals (n = 145), 55.8% (81/145) were septic and 44.2% (64/145) were SNS. In addition, 43.4% (63/145) and 56.6% (82/145) of the hospitalized foals were classified as NMS and sick non‐NMS, respectively. Blood culture was performed in 69 septic foals, of which 63.8% (44/69) were positive. Within NMS foals, 61.9% (39/63) were septic and 38.1% (24/63) were SNS foals. In the sick non‐NMS group, 51.2% (42/82) were septic and 48.8% (40/82) SNS foals. Proportions of septic and SNS foals between NMS and sick non‐NMS were not statistically different (*P* = .20). Healthy foals (n = 80) comprised 45 fillies, 25 colts, and 10 unknowns. Hospitalized foals comprised 58 fillies, 80 colts, and 7 unknowns. The proportion of colts to fillies was higher in hospitalized than in healthy foals (*P* = .0005). The median age at first sampling for healthy foals was 24 hours (1‐72 hours) and for hospitalized foals was 9 hours (1‐72 hours), which were not statistically different (*P* = .22). Hormone concentrations between sex within groups were not statistically different.

Healthy foals comprised Thoroughbreds (n = 58), Standardbreds (n = 19), American Paint Horses (n = 2), and Quarter Horse (n = 1). Hospitalized foals included Thoroughbreds (n = 121), Standardbreds (n = 9), Quarter Horses (n = 5), Saddlebreds (n = 5), Belgians (n = 2), Warmbloods (n = 2), American Paint Horse (n = 1), and Arabian (n = 1).

The survival rate in hospitalized foals was 75.2% (109/145). Forty‐nine (61.2%) septic and 60 (93.7%) SNS foals survived, and this proportion was statistically different (*P* < .01). Sixty‐two (74.6%) sick non‐NMS and 47 (75.8%) NMS foals survived, and this proportion was not statistically different (*P* = .91). Of the 36 nonsurviving foals, 27.8% (10/36) died between admission and 24 hours, 19.4% (7/36) between 24‐48 hours, and 11.1% (4/36) between 48‐72 hours.

### 3.2 Androgen concentrations

**FIGURE 1:**
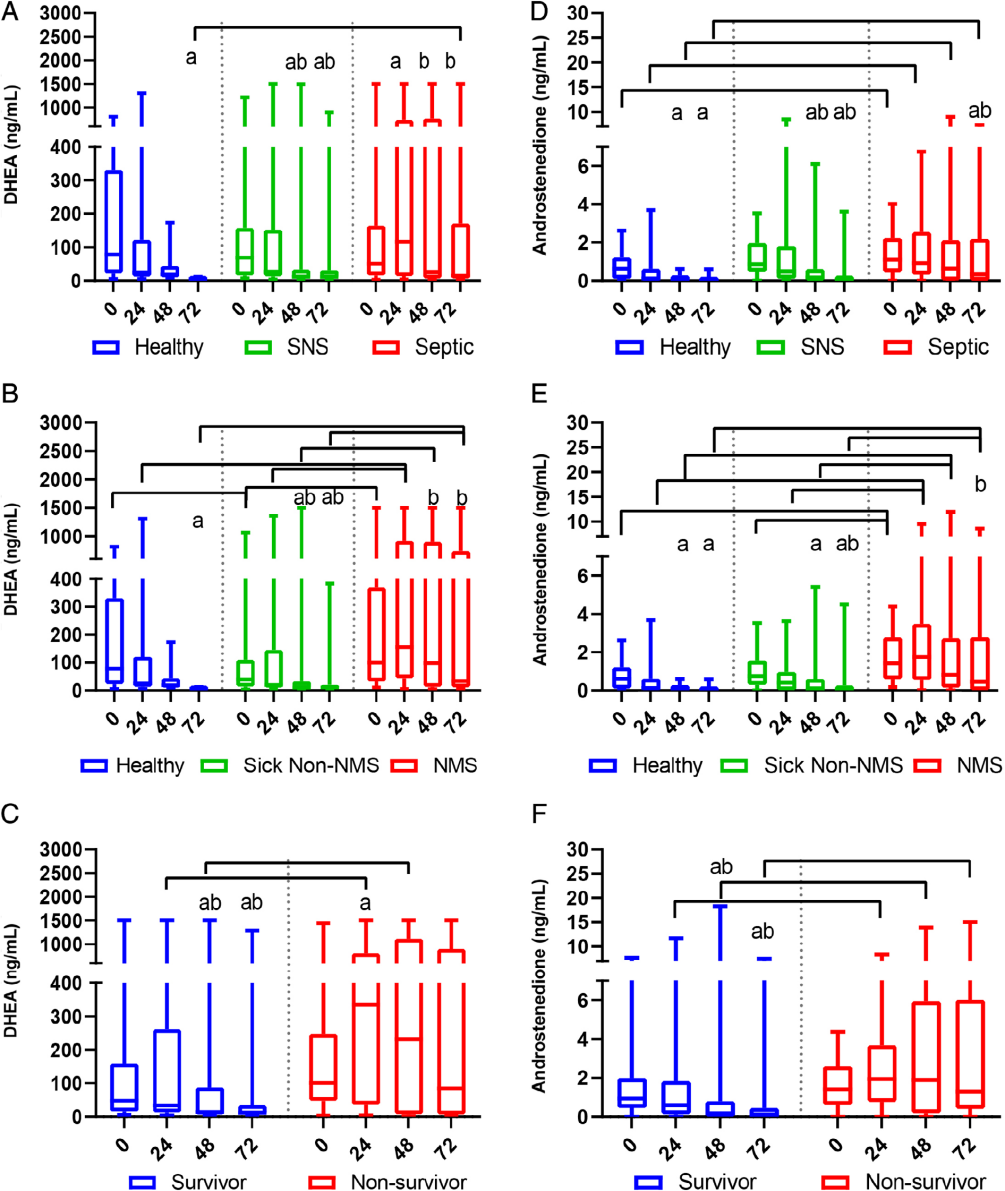
Serum DHEA and androstenedione concentrations in healthy and hospitalized foals over time. Values are expressed as medians with inner quartile ranges; whiskers represent the 95% ranges of the data. Serum DHEA concentrations in healthy (n = 80), SNS (n = 64), and septic foals (n = 81) (A), in healthy (n = 80), sick non‐NMS (n = 63), and NMS (n = 82) foals (B), and in surviving (n = 109) and nonsurviving (n = 36) foals (C). Serum androstenedione concentrations in healthy (n = 80), SNS (n = 64), and septic foals (n = 81) (D), in healthy (n = 80), sick non‐NMS (n = 63), and NMS (n = 82) foals (E), and in surviving (n = 109) and nonsurviving (n = 36) foals (F). Brackets indicate significant differences between groups at the indicated time point (*P* < .05); “a” indicates significantly different from time zero (admission) within group; “b” indicates significantly different from 24 hours within group. Letters of significance denote *P* < .05. DHEA, dehydroepiandrosterone; NMS, neonatal maladjustment syndrome; SNS, sick nonseptic

Serum DHEA concentrations were not significantly different at admission, 24, or 48 hours among healthy, SNS, and septic foals. However, at 72 hours, septic foals had a significantly higher DHEA compared to healthy foals (Figure 1A; Supplementary Table 1; *P* = .02). NMS foals had higher DHEA concentrations than sick non‐NMS foals at all time points (*P* < .003) and healthy foals at 0, 24, and 72 hours (Figure 1B; Supplementary Table 1; *P* < .05). Nonsurviving foals had higher DHEA at 24 and 48 hours than surviving foals (Figure 1C; Supplementary Table 2; *P* < .05). Apart from nonsurviving foals, DHEA concentrations decreased over time among all foal groups (Figures 1A‐C; Tables 1 and 2; *P* < .05).

**TABLE 1:** Odds ratios for nonsurvival at single time points. Referent for odds ratios is upper limit of 95% CI from healthy foals

Variable	Time	Range	OR	95% CI	*P* value
DHEA (ng/mL)	24	>33.2	3.6	1.22‐10.6	.02
		≤33.2	Referent		
	48	>37.1	5.9	1.88‐18.8	.002
		≤37.1	Referent		
	72	>18.8	3.6	1.07‐12.1	.04
		≤18.8	Referent		
Testosterone (ng/dL)	0	>12.3	3.3	1.37‐8.1	.008
		≤12.3	Referent		
	72	>5.01	3.92	1.1‐13.6	.03
		≤5.01	Referent		
DHT (pg/mL)	72	>680	3.68	1.05‐12.9	.04
		≤680	Referent		

Abbreviations: DHEA, dehydroepiandrosterone; DHT, dihydrotestosterone.

**TABLE 2:** Odds ratios for nonsurvival in foals with negative delta values. Referent for odds ratios is clearance of steroid (Δ > 0)

Variable	Time interval	Range	OR	95% CI	*P* value
DHEA (ng/mL)	0‐24	≤0	4.13	1.3‐13.2	.02
		>0	Referent		
Androstenedione (ng/mL)	0‐24	≤0	7	2.1‐22.5	.001
		>0	Referent		
	24‐48	≤0	3.3	1.04‐10.6	.04
		>0	Referent		
	0‐72	≤0	4.1	1.1‐15	.03
		>0	Referent		
DHT (pg/mL)	0‐24	≤0	6.4	1.3‐29.9	.02
		>0	Referent		
	48‐72	≤0	7.1	1.3‐36.3	.02
		>0	Referent		
	0‐72	≤0	4.4	1.04‐18.9	.04
		>0	Referent		

Abbreviations: DHEA, dehydroepiandrosterone; DHT, dihydrotestosterone.

Serum androstenedione concentrations were higher at all time points in septic and NMS compared to healthy and sick non‐NMS foals (Figures 1D,E; Supplementary Table 1; *P* < .01). Androstenedione concentrations did not differ between surviving and nonsurviving foals at admission, but were higher in nonsurviving foals at later time points (Figure 1F; Supplementary Table 2; *P* < .01). Excluding nonsurviving foals, androstenedione concentrations decreased over time among all foal groups (Figures 1D‐F; Tables 1 and 2; *P* < .05).

**FIGURE 2:**
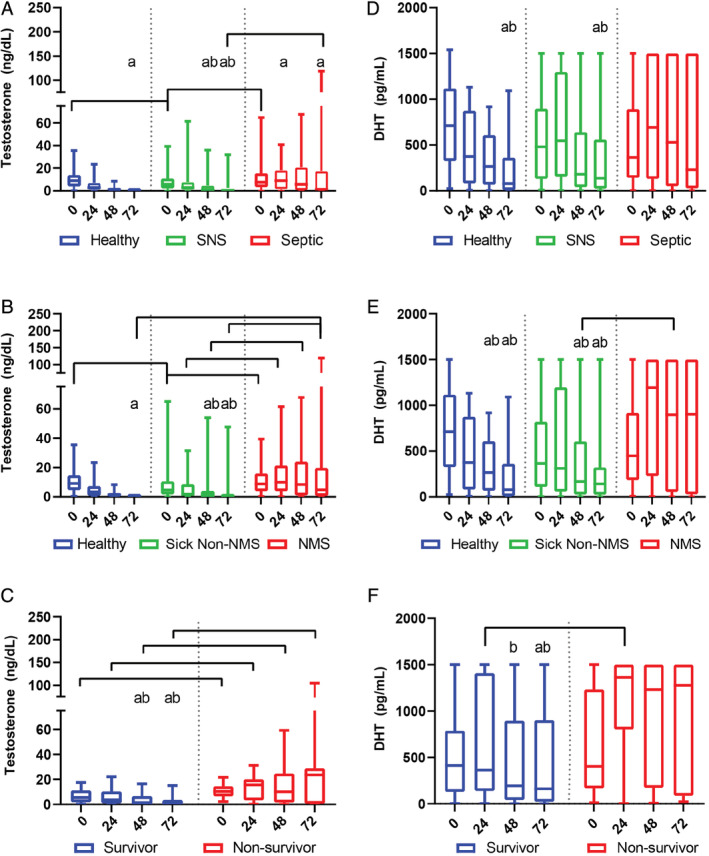
Serum testosterone and DHT concentrations in healthy and hospitalized foals over time. Values are expressed as medians with inner quartile ranges; whiskers represent the 95% ranges of the data. Serum testosterone concentrations in healthy (n = 80), SNS (n = 64), and septic (n = 81) foals (A), in healthy (n = 80), sick non‐NMS (n = 63), and NMS (n = 82) foals (B), and in surviving (n = 109) and nonsurviving (n = 36) foals (C). Serum DHT concentrations in healthy (n = 80), SNS (n = 64), and septic (n = 81) foals (D), in healthy (n = 80), sick non‐NMS (n = 63), and NMS (n = 82) foals (E), and in surviving (n = 109) and nonsurviving (n = 36) foals (F). Brackets indicate significant differences between groups at the indicated time point (*P* < .05); “a” indicates significant difference from zero (admission) within group; “b” indicates significant difference from 24 hours within group. Letters of significance denote *P* < .05. DHT, dihydrotestosterone; NMS, neonatal maladjustment syndrome; SNS, sick nonseptic

Serum testosterone concentrations were higher in septic compared to SNS foals at admission and 72 hours (Figure 2A; Supplementary Table 1; *P* < .05). Serum testosterone concentrations decreased over time in healthy, SNS, sick non‐NMS and septic foals (Figures 2A,B; Supplementary Table 1; *P* < .05), but remained elevated in NMS and nonsurviving foals (Figures 2B,C; Tables 1 and 2). Serum testosterone concentrations were higher at all time points in nonsurviving compared to surviving foals (Figure 2C; Supplementary Table 2; *P* < .02).

Serum DHT concentrations were not different among healthy, SNS, and septic foals at any time point (Figure 2D; Supplementary Table 1). Similarly, serum DHT concentrations were not different between healthy, sick non‐NMS, or NMS foal groups at admission, 24, or 72 hours, but were higher at 48 hours in NMS compared to sick non‐NMS foals (Figure 2E; Supplementary Table 1; *P* = .009). Nonsurviving foals had higher serum DHT concentrations than survivors at 24 hours (Figure 2F; Supplementary Table 2; *P* = .022). Serum DHT concentrations decreased over time in healthy, SNS, and sick non‐NMS foals (*P* < .05), but did not in septic, NMS, and nonsurviving foals (Figures 2D‐F; Tables 1 and 2).

### 3.3 Androgen clearance and percentage change over time

**FIGURE 3:**
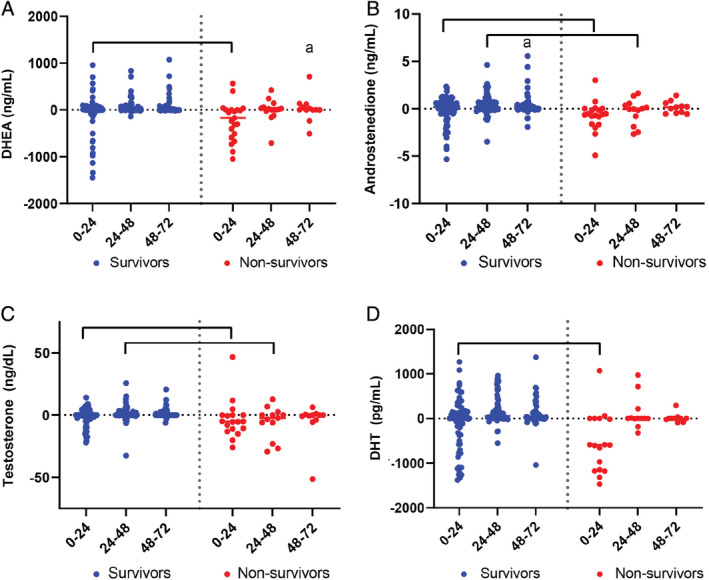
Delta androgen values in surviving (n = 109) and nonsurviving (n = 36) foals over time. Values are expressed as individual data points. Delta values for DHEA (A), testosterone (B), androstenedione (C), and DHT concentrations (D). Brackets indicate significant differences between groups at the indicated time point (*P* < .05); “a” indicates significant difference from 0 to 24 within group (*P* < .05). DHEA, dehydroepiandrosterone; DHT, dihydrotestosterone

**FIGURE 4:**
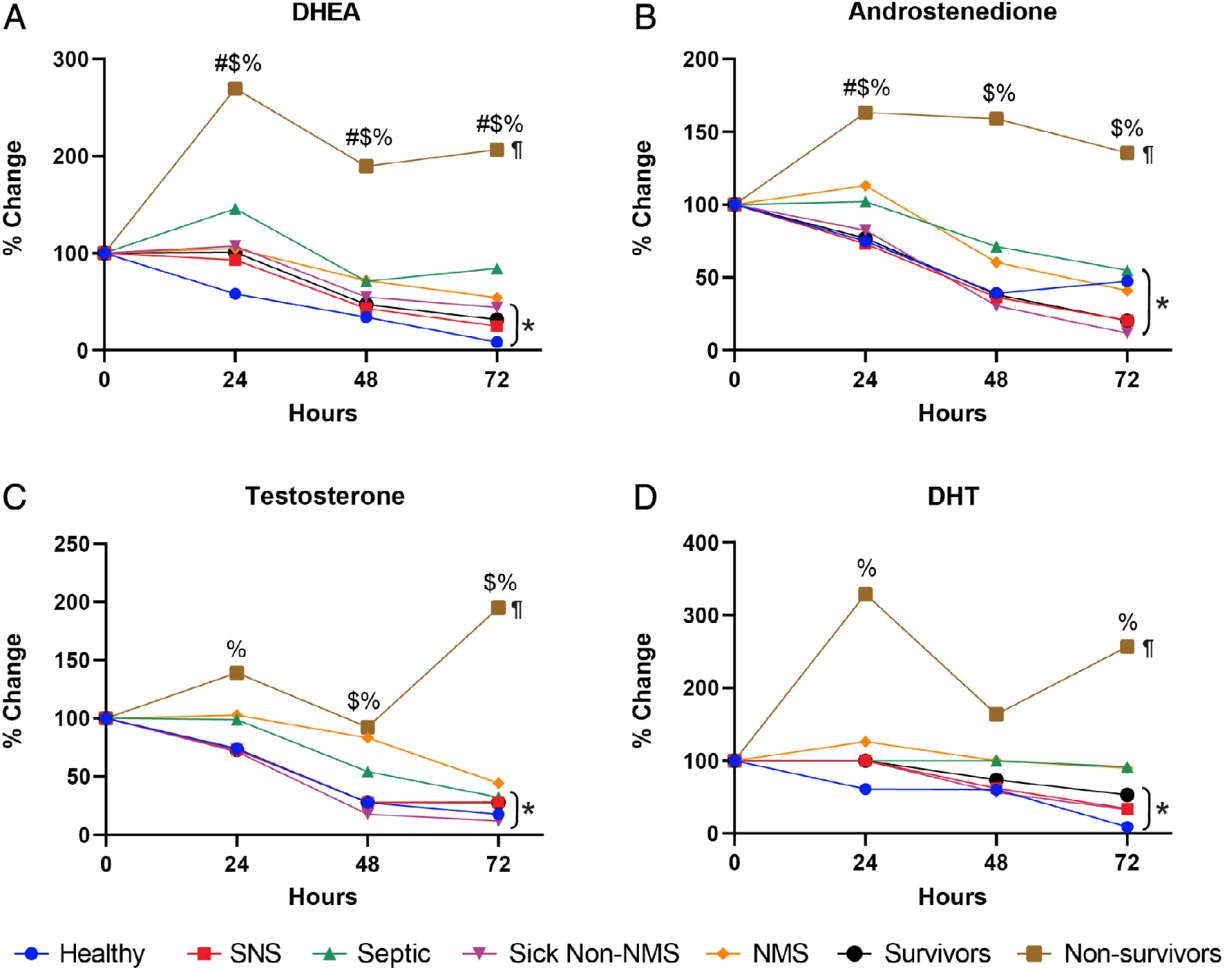
Percentage change in androgen concentrations over time based on baseline values (100%). Values are expressed as medians. Percentage values for DHEA (A), testosterone (B), androstenedione (C), and DHT (D) in healthy (blue circles), SNS (red squares), septic (green triangles), sick non‐NMS (purple down triangles), NMS (orange diamonds), surviving (black circles), and nonsurviving (brown squares) foals. # indicates significant difference among healthy, SNS and septic foals; $ indicates significant difference among healthy, sick non‐NMS, and NMS foals; % indicates significant difference between survivors and nonsurvivors; * indicates a significant decrease from baseline in bracketed groups; ¶ indicates significant increase from baseline. Symbols of significance denote *P* < .05. DHEA, dehydroepiandrosterone; DHT, dihydrotestosterone NMS, neonatal maladjustment syndrome; SNS, sick nonseptic

Septic foals had less DHEA clearance (Δ_0‐24_) than healthy foals (Supplementary Table 3; *P* < .05). Clearance among healthy foals was significantly greater at earlier time points (Δ_0‐24_ vs Δ_24‐48_), whereas septic foals had higher DHEA clearance at later time points (Δ_24‐48_ vs Δ_0‐24_) (Supplementary Table 3; *P* < .03). Nonsurviving foals had decreased DHEA clearance (Δ_0‐24_) (Figure 3A; Supplementary Table 4; *P* = .004). Median percentage DHEA concentrations from baseline to 72 hours decreased to 8% to 84% among healthy, SNS, septic, sick non‐NMS, NMS, and surviving foals, but increased to 207% in nonsurviving foals (Figure 4A; *P* < .05).

Serum androstenedione clearance (Δ_0‐24_) was significantly lower in septic foals than healthy foals (Supplementary Table 3; *P* < .05). Androstenedione clearance in SNS foals was greater at earlier time points (Supplementary Table 3; *P* < .05). The androstenedione clearance (Δ_0‐24_, Δ_24‐48_) was significantly lower in nonsurviving than surviving foals (Figure 3B; Supplementary Table 4; *P* < .04). From baseline to 72 hours, the median percentage change in androstenedione concentrations decreased to 20% to 55% among healthy, SNS, septic, sick non‐NMS, NMS, surviving foals, but increased to 136% in nonsurviving foals (Figure 4B; *P* < .05).

Serum testosterone clearance did not differ between healthy and septic foals, but did between septic and SNS foals (Supplementary Table 3; *P* < .02). Nonsurviving foals had lower testosterone clearance (Δ_0‐24_, Δ_24‐48_) than surviving foals (Figure 3C; Supplementary Table 4; *P* < .05). The median percentage change in testosterone concentrations decreased from baseline to 72 hours to 18% to 44% in healthy, SNS, septic, sick non‐NMS, NMS, and surviving foals whereas in nonsurviving foals increased to 195% (Figure 4C; *P* < .05).

Serum DHT clearance (Δ_0‐24_) was lower in septic than healthy foals (Supplementary Table 3; *P* < .05). Clearance in healthy foals was higher at earlier time points (Supplementary Table 3; *P* < .01). Serum DHT clearance (Δ_0‐24_) was significantly lower in nonsurvivors compared to survivors (Figure 3D; Supplementary Table 4; *P* < .001). From baseline to 72 hours, median percentage DHT concentrations decreased to 9% to 53% among healthy, SNS, sick non‐NMS and surviving foals, with minimal changes in septic and NMS foals, but increased in nonsurviving foals to 257% (Figure 4D; *P* < .05).

### 3.4 ACTH and ACTH:androgen ratios

Plasma ACTH concentrations at admission were higher in septic and NMS than healthy foals (Supplementary Table 1; *P* < .001). Plasma ACTH concentrations were not different when comparing healthy to SNS and sick non‐NMS foals at any time point. Plasma ACTH concentrations were not different in surviving and nonsurviving foals at admission or 48 hours, but were higher in nonsurvivors at 24 hours (*P* = .04). Over time, ACTH concentrations decreased in NMS foals (Supplementary Table 1; *P* = .007), but did not change significantly in other groups. ACTH concentrations decreased in all hospitalized foals, but were not statistically different than time 0.

The ACTH:androgen ratios are listed in Supplementary Table 5. At admission, the ACTH:DHEA ratio was significantly higher in septic, SNS, NMS, and sick non‐NMS compared to healthy foals (*P* < .01). The ACTH:DHEA ratio increased over time in healthy foals (*P* = .002), whereas no change was seen among other groups.

The ACTH:androstenedione ratio was not different among foal groups upon admission. However, the ratio was significantly lower in NMS compared to healthy foals at 48 hours (*P* = .03). Healthy foals experienced a significant increase from admission to 48 hours (*P* < .01). SNS and NMS foals had a significant decrease in the ACTH:androstenedione ratio from 0 to 24 hours (*P* < .04).

The ACTH:testosterone ratio was significantly higher in septic and NMS compared to healthy foals upon admission (*P* < .02), but not in SNS or sick non‐NMS foals. NMS foals had significantly higher ACTH:testosterone ratios at admission compared to 24 and 48 hours (*P* < .05).

At admission, the ACTH:DHT ratio was higher in septic and NMS compared to healthy foals (*P* = .02). NMS foals had a significantly lower ACTH:DHT ratios at 24 hours than at admission (*P* < .05).

No differences in ACTH:steroid ratios between survivors and nonsurvivors were found for any of the steroids measured, at any time point.

### 3.5 Odds ratios for nonsurvival

Results of univariate analysis for survival are listed in Table 1. Hospitalized foals with serum DHEA concentrations >32.1 ng/mL at 24 hours, >37.1 ng/mL at 48 hours, and >18.8 ng/mL at 72 hours were >3.6 times more likely to die (Table 1; *P* < .04). Hospitalized foals with serum testosterone concentrations >12.31 ng/mL at admission and >5 ng/mL at 72 hours were >3 times more likely to die (Table 1; *P* < .05). Foals with serum DHT concentrations >680 pg/mL at 72 hours were >3.6 times more likely to die (Table 1; *P* = .04). Androstenedione concentrations were not associated with mortality at any time point.

Table 2 shows odds ratios for nonsurvival in foals with steroid Δ values of ≤0. Foals with Δ_0‐24_ values ≤0 (minimal clearance) for DHEA, androstenedione, and DHT were >4 times more likely to die (Table 2; *P* < .05). In addition, foals with a Δ_24‐48_ ≤ 0 for androstenedione and DHT, a Δ_48‐72_ ≤ 0 for DHT, and a Δ_0‐72_ ≤ 0 for androstenedione and DHT had a >3.3 times higher chance of mortality (Table 2; *P* < .05).

## 4 DISCUSSION

This study shows that increased serum androgen concentrations, similar to progestogens, are associated with disease severity and outcome in hospitalized neonatal foals. Healthy foals showed a steady decrease in serum androgen concentrations over the study period that was not evident in severely affected or nonsurviving foals. These results indicate that steroid dynamics in the immediate postpartum period are linked to organ function and viability, which have clinical and prognostic implications.

Progestogens, androgens, and estrogens play key roles in pregnancy establishment, maintenance and termination, as well as fetal tissue differentiation and maturation.^3^, ^8^, ^10^ Pregnenolone (progestogen) and DHEA (androgen) are the main steroid precursors in the equine fetus and their sources are the adrenal glands and fetal gonads.^8^, ^26^, ^27^ These steroids are transported to the placenta to be further metabolized into progestogens (dihydroprogesterone [DHP], allopregnanolone), androgens (androstenedione, testosterone, DHT), and estrogens (estrone, 17β‐estradiol, equilin).^28^, ^29^ Some of these steroid metabolites are transported to maternal circulation (eg, DHP, 20α‐hydroxy‐5α‐pregnane‐3‐1 [a DHP metabolite]), whereas others are transported to the fetus (eg, progesterone, DHP).^9^

Steroid concentrations in the newborn foal reflect placental as well as fetal adrenocortical and gonadal steroidogenic activity.^30^ In these foals, glucocorticoids, mineralocorticoids, progestogens, and androgens are cleared rapidly from circulation.^11^, ^12^, ^13^ This was evident for androgens in the blood of the healthy foals of our study, where DHEA, androstenedione, testosterone, and DHT concentrations decreased steadily. However, in critically ill foals, serum androgens remained elevated over the study period. This was supported by the low steroid Δ values and percent changes, indicating decreased clearance that was also linked to mortality.

The delayed decrease in serum androgen concentrations, which was more evident in critically ill and nonsurviving foals, suggests that impaired clearance rather than increased secretion is central to these abnormal steroid patterns. These findings could be reflecting a combination of impaired hepatic processing, altered protein binding, and reduced renal excretion. We speculate that impaired steroid metabolism and excretion are the main reasons because concentrations and time to decrease for most androgens were associated with disease severity. Pathologic processes specific to steroid metabolism in foals remain to be investigated. Hepatic or renal dysfunction leading to failure to metabolize or excrete steroids was proposed as potential explanations for increased progestogens in premature foals.^31^ In other species, the liver is the main site of steroid catabolism, where they are processed from lipophilic compounds through reduction, hydroxylation, aromatization, and conjugation (sulfation, glucuronidation) into less toxic hydrophilic metabolites to facilitate their biliary and urinary excretion.^32^ In rodents and people, androgen clearance occurs in the liver.^33^ People with hepatic disease have reduced cortisol clearance^32^ and critically ill human patients have decreased elimination of cortisol.^34^

In this study, we used delta values to evaluate the dynamics of androgen concentrations in blood because they are easy to calculate, can be generated with 2 time points, and provide information in short time segments. We also calculated percentage changes to adjust concentrations to baseline values to facilitate result interpretation. Calculation of steroid half‐lives could have been an alternative method to evaluate clearance. However, around half of nonsurviving foals did not live beyond 2 sampling points and a quarter did not have delta values because of mortality after initial sampling. Thus, determination of half‐lives would have been challenging because this calculation requires 3 or more time points and steroid half‐lives may have been biased to foals that lived longer. Based on the higher delta values and percentage change from baseline in critically ill compared to healthy foals, one would predict that that the sicker foals would have longer steroid half‐lives.

Recent work found that cortisol and progesterone concentrations remained elevated in sick foals born to mares with experimental placentitis.^13^ In this study, we showed that similar to people, steroid concentrations remain elevated in foals in response to critical illness. Decreased hepatic catabolism is a reasonable explanation for elevated androgen concentrations in the blood of neonatal foals in this study. Liver disease and subclinical hepatic injury with impaired function occur in sick foals.^35^ Hospitalized foals often have increased hepatic enzyme activities, which is underplayed and assumed to be a consequence of systemic inflammation or endotoxemia.^35^ To the authors' knowledge, studies on steroid clearance in healthy or diseased foals have not been done. An alternative, but not exclusive theory could be that decreased steroid clearance is a compensatory mechanism for low adrenocortical steroid production in foals with RAI.

Another question that remains unanswered is what are the consequences of increased serum androgen concentrations in the sick equine neonate. In our study, elevated androgen concentrations were associated with nonsurvival, which could potentially contribute to disease progression since androgens have immunosuppressive effects. Testosterone suppresses T‐cell immune response via IL‐10 and TGF‐β.^36^, ^37^ Although the cytokine gene expression of T‐cells has not been investigated in septic foals, they are known to have altered cytokine profiles.^38^, ^39^ This area might warrant further investigation, however, the role of androgens in the face of sepsis could have a much deeper role than just modulation of Regulatory T‐cell cytokine production.

Because of their lipophilic nature, the majority of steroids in circulation are bound to transport proteins. Cortisol circulates free and bound to cortisol binding globulin (CBG) and albumin. Critically ill foals have increased cortisol concentrations,^15^, ^18^, ^25^ and most assays used in these studies measured total cortisol concentrations. Hart et al^40^ showed that septic foals have increased free cortisol concentrations. Sex hormone binding globulin (SHBG) is a major transporter for sex steroids. In people, 1% to 2% of total testosterone is free, whereas the rest is protein bound.^41^ Similar to CBG, SHBG is produced by the liver and affected by diseases; in people, SHBG is negatively correlated with sepsis severity.^42^ Thus, one could infer that decreased concentrations of SHBG in foals could impact their bioavailability and clearance. In human patients, high concentrations of free 17β‐estradiol (an androgen metabolite) can predict mortality.^43^ Although information in SHBG is lacking in foals, hypoalbuminemia is a frequent finding, especially in septic foals. Although we did not measure free fractions of androgens in blood, it is likely that alterations in protein binding during illness affect their concentrations and bioavailability. Future investigation in this area in newborn foals may be warranted.

In our study, serum androgen concentrations were higher in NMS compared to healthy and sick non‐NMS foals, which is a pattern similar to progestogens.^15^, ^17^ The diagnosis of NMS can be controversial and subjective as it is currently a clinical diagnosis.^24^ It can be difficult to differentiate from sepsis and at times can occur concurrently with sepsis. Our analysis was likely affected by the inclusion of NMS foals with sepsis; however, NMS foals without sepsis still had higher androgen concentrations than SNS foals without the clinical diagnosis of NMS. Although the overlap between septic and NMS foals cannot be ignored and the reader must interpret these results with caution, the author's still believe that this study indicates that serum androgen concentrations and the perinatal steroid balance could potentially contribute to the pathogenesis of NMS and other disorders in newborn foals, including dysmaturity and sepsis. Androgens interact with membrane receptors in neurons and glial cells. For example, although progesterone and allopregnanolone are GABA_A_ receptor agonists, DHEA is an antagonist,^44^, ^45^ which could influence disease development and progression in the equine neonate. DHEA‐sulfate (DHEA‐S) is a metabolite of DHEA that is a more potent GABA_A_ inhibitor than DHEA.^45^ DHEA also has positive allosteric activity at NMDAR, which mediate excitotoxicity,^20^ and may play a role in NMS.^24^ It has been suggested that the DHEA and DHEA‐S balance may be critical for neuromodulation.^20^

Testosterone has pro‐ or anticonvulsant effects, which are attributable to its metabolism to other androgens or aromatization to estrogens.^21^ Metabolism to 17β‐estradiol reduced the seizure threshold, whereas conversion to DHT and androstanediol had anticonvulsant activity.^21^ Testosterone is converted to DHT, which is reduced to androstanediol^17^ in a similar process by which progesterone is converted to allopregnanolone, a progestogen known to induce NMS behavior in foals.^15^ This opens the question as to whether the sex steroid balance is more important than absolute concentrations on neurological disorders in newborn foals.

Elevated ACTH concentrations could have increased androgen concentrations in the sick foals of this study. However, that seems less likely based on differing longitudinal changes in ACTH and androgen concentrations between healthy and sick foals. Similar to a previous study,^46^ healthy foals had a slight increase in ACTH concentrations with a decline in androgens over time, suggesting that postnatal shifts in steroidogenesis, reduction in adrenocortical sensitivity and cortisol secretion, and removal of placental steroids are at play. On the contrary, in sick foals, serum androgen concentrations remained elevated while ACTH concentrations decreased. Moreover, ACTH did not differ between groups at later time points. This indicates that reduced clearance is a more feasible explanation for high androgen concentrations in the hospitalized foals of this study.

The ACTH:cortisol is a practical tool that has been used to assess hypothalamic‐pituitary‐adrenal axis activity and adrenocortical insufficiency (RAI/CIRCI) in hospitalized foals.^18^, ^47^ Because ACTH stimulation induces androgen steroidogenesis,^4^ it is not surprising that illness alters the ACTH:androgen ratios.^15^ ACTH has been evaluated in reference to other steroids (progestogens and androgens) in healthy and sick foals.^15^ In the healthy foals of this study, the ACTH:androgen ratio was low at initial sampling and rose over time, similar to trends documented for the ACTH:cortisol ratio.^46^, ^47^ The high ACTH:androgen ratios in septic foals at upon admission were comparable to previous findings.^15^ We were unable to demonstrate a difference between ACTH:androgen ratios in survivors and nonsurvivors; this combined with the lack of a difference between healthy and sick foals at later time points, makes clinical interpretation and use of the ACTH:androgen ratio difficult.

One potential limitation of this study was the use of the sepsis score to categorize foals, which is known to have poor sensitivity and predictive value.^48^, ^49^, ^50^ However, this score is 1 of the few tools to assess disease severity in hospitalized foals. Another limitation could be the use of immunoassays to measure selected androgens instead of methods such as liquid chromatography mass spectrometry (LC/MS) that can measure multiple steroids in a single sample. However, immunoassays used in this study have been validated for equine samples, we are familiar with these techniques, and steroid profiling was not the goal of this study. In addition, immunoassays are the routine method for steroid measurement in the practice setting. Using LC/MS would have provided a more detailed androgen profile; however, it also has its limitations, including cost per sample, equipment, expertise, and turnaround time.

## 5 CONFLICT OF INTEREST DECLARATION

Authors declare no conflict of interest.

## OFF‐LABEL ANTIMICROBIAL DECLARATION

Authors declare no off‐label use of antimicrobials.

## INSTITUTIONAL ANIMAL CARE AND USE COMMITTEE (IACUC) OR OTHER APPROVAL DECLARATION

Approved by The Ohio State University IACUC. This was not an interventional study. Blood samples collected from healthy and sick foals were collected with owner consent.

## HUMAN ETHICS APPROVAL DECLARATION

Authors declare human ethics approval was not needed for this study.

## Supporting information


**Supplementary Table 1** Androgen and ACTH concentrations in healthy and hospitalized foals over time. Data expressed as medians and ranges.
**Supplementary Table 2**: Androgen concentrations in surviving and non‐surviving foals over time. Data expressed as medians and ranges.
**Supplementary Table 3**: Androgen delta values in healthy, SNS, and septic foals. . Data expressed as medians and ranges.
**Supplementary Table 4**: Androgen delta values in surviving and non‐surviving foals. Data expressed as medians and ranges.
**Supplementary Table 5**: ACTH:androgen ratios in healthy and hospitalized foals over time. Data expressed as median and ranges.
