# Supplementary material for: Dynamics of androgens in healthy and hospitalized newborn foals
Source: J Vet Intern Med. 2020 Dec 5;35(1):538–49. doi: 10.1111/jvim.15974 (PMC7848305; doi:10.1111/jvim.15974)
Supplement: Supplementary file 1 — Supplementary Table 1 Androgen and ACTH concentrations in healthy and hospitalized foals over time. Data expressed as medians and ranges. Supplementary Table 2: Androgen concentrations in surviving and non‐surviving foals over time. Data expressed as medians and ranges. Supplementary Table 3: Androgen delta values in healthy, SNS, and septic foals. . Data expressed as medians and ranges. Supplementary Table 4: Androgen delta values in surviving and non‐surviving foals. Data expressed as medians and ranges. Supplementary Table 5: ACTH:androgen ratios in healthy and hospitalized foals over time. Data expressed as median and ranges. [file JVIM-35-538-s001.pdf]

SUPPLEMENTARY TABLE 1: Androgen and ACTH concentrations in healthy and hospitalized foals over time. Data expressed as medians and ranges.

DHEA (ng/mL)	**Healthy (n = 80)**	**SNS (n = 64)**	**Septic (n = 81)**	**Sick non-NMS (n = 63)**	**NMS (n = 82)**
0	78.4 (4.23-1152)	68.5 (6.29-1500)	50.9 (3.15-1500)	39.4 (3.15-1415)	100.3 (3.57-1500)[#$]
24	24.8 (5.26-1500)	26.9 (4.27-1500)	115.9 (3.60-1500)[a]	19.5 (3.60-1500)	156 (4.20-1500)[#$]
48	18.8 (8.85-173.6)	11.5 (3.01-1500)[ab]	26.1 (2.37-1500)[b]	9.57 (2.37-1500)[ab]	0.94 (4.16-1500)[$b]
72	5.62 (2.55-11.4)[a]	10.8 (3.90-1500)[ab]	15.7 (2.35-1500)[#b]	9.72 (2.35-1500)[ab]	0.47 (5.04-1500[)#$b]
Androstenedione (ng/mL)					
0	0.61 (0.03-3.85)	0.87 (0.03-0.623)	1.10 (0.03-7.68)[#]	0.75 (0.03-4.37)	1.42 (0.03-7.68) [#$]
24	0.12 (0.03-4.64)	0.50 (0.03-10.2)	0.92 (0.03-10.2)[#]	0.39 (0.03-4.59)	1.80 (0.03-10.2)[#$]
48	0.03 (0.03-0.62[)a]	0.16 (0.03-8.27)[ab]	0.63 (0.03-10.2)[#]	0.03 (0.03-6.95)[a]	0.94 (0.03-10.2)[#$]
72	0.03 (0.03-0.60)	0.05 (0.03-7.68)[ab]	0.35 (0.03-10.2)[#ab]	0.03 (0.03-6.79)[ab]	0.47 (0.03-10.2)[#$b]
Testosterone (ng/dL)					
0	9.50 (0.35-35.4)	6.03 (0.11-39.3)[#]	6.38 (0.23-64.9)[@]	4.70 (0.10-64.9)[#]	8.76 (0.23-39.3)[$]
24	2.78 (0.11-23.3)	3.31 (0.17-61.5)	6.80 (0.001-40.9)	2.3 (0.001-31.5)	9.89 (0.18-61.5)[$]
48	1.3 (0.08-8.30)	1.02 (0.003-35.9)[ab]	4.06 (0.015-67.6) a	1.04 (0.012-53.8)[ab]	8.41 (0.003-67.6)[$]
72	0.48 (0.03-0.73)[a]	1.87 (0.01-31.9)[ab]	2.6 (0.001-119)[@a]	0.62 (0.001-47.5)[ab]	4.74 (0.01-119)[#$]
DHT (pg/mL)					
0	710.6 (24.8-1539)	478.7 (6.59-2181)	364.6 (1.96-2661)	364.6 (1.96-2390)	447.3 (6.94-2661)
24	374.5 (5.62-1131)	545.4 (1.94-2681.3)	692.0 (1.78-2778)	310.6 (1.80-2352)	1193 (1.94-2778)
48	264.0 (2.69-916.2)	180.9 (1.03-2814)	529.0 (0.29-2875)	167.6 (0.29-28.75)	896.1 (1.03-2814)[$]
72	77.1 (1.03-1090)[ab]	136.0 (1.09-2066)[ab]	229.1 (0.32-3402)	1416 (0.80-3005) [ab]	900.3 (0.32-3401.6)
ACTH (pg/mL)					

0	24.4 (10-55.6)	42.9 (10-748)	112 (10-988)[#]	51.3 (10-961)	163 (23.2-988)[#]
24	21.6 (10-53.4)	33.6 (10-933)	48.5 (10-615)	43.9 (10-933)	49.5 (10-437)[a]
48	34.5 (17.9-56.8)	23.2 (16-177)	34.2 (10-568)	22.5 (10-177)	47 (10-568)[a]

# indicates significant difference from healthy foals; @ indicates significance from SNS foals; $ indicates significant difference from sick non-NMS foals; “a” indicates significant difference from time zero within group; “b” indicates significant difference from time 24 within group. Symbols and letters of significance denote $P < .05$.

Abbreviations: DHEA; dehydroepiandrosterone; DHT, dihydrotestosterone; SNS, sick non-septic; NMS, neonatal maladjustment syndrome.

SUPPLEMENTARY TABLE 2: Androgen concentrations in surviving and non-surviving foals over time. Data expressed as medians and ranges.

DHEA (ng/mL)	**Survivor (n = 109)**	**Non-survivor (n = 36)**
0	49.0 (3.28-1500)	101 (3.15-1500)
24	33.9 (3.60-1500)	335 (4.19-1500) [%a]
48	14.1 (3.01-1500)[ab]	232 (2.37-1500)%
72	12.2 (2.35-1500)[ab]	85.0 (3.28-1500)
Androstenedione (ng/mL)		
0	0.94 (0.03-7.69)	1.40 (0.03-4.37)
24	0.61 (0.03-10.2)	1.94 (0.03-8.29)[%]
48	0.17 (0.03-10.2)[ab]	1.90 (0.03-10.2)[%]
72	0.03 (0.03-7.42)[ab]	1.30 (0.03-10.2)[%]
Testosterone (ng/dL)		
0	5.6 (0.10-39.3)	10.4 (0.12-64.9)[%]
24	3.7 (0.001-61.5)	15.7 (0.10-40.9)[%]
48	1.3 (0.003-61.7)[ab]	10.0 (0.20-67.6)[%]
72	0.8 (0.001-53.8)[ab]	23.6 (0.04-119)[%]
DHT (pg/mL)		
0	412 (1.96-2661)	402 (13.2-2390)
24	364 (1.80-2778)	1363 (2.27-2352)[%]
48	193 (0.29-2814)[b]	1257 (8.01-2875)
72	162 (0.32-3402)[ab]	1348 (22.3-3005)
ACTH (pg/mL)		
0	53.5 (10-998)	420 (25.7-961)
24	38.2 (10-220)	437 (10-993)[%]
48	23.8 (10-391)	289 (10-568)

% indicates significant difference from surviving foals; "a" indicates significant difference from time zero within group; "b" indicates significant difference from time 24 within group. Symbols and letters of significance denote $P < .05$.

Abbreviations: DHEA; dehydroepiandrosterone; DHT, dihydrotestosterone.

SUPPLEMENTARY TABLE 3: Androgen delta values in healthy, SNS, and septic foals. . Data expressed as medians and ranges.

Δ DHEA (ng/mL)	**Healthy (n = 80)**	**SNS (n = 64)**	**Septic (n = 81)**
0-24	14.4 (-348 - 568)	0.90 (-16.5 - 1075)	-5.6 (-1449 – 958)[#]
24-48	5.2 (-11.5 - 386)[a]	10.2 (-26.0 – 835)	10.0 (-707 – 713)[a]
48-72	8.9 (6.5 – 15.1)	0 (-16.5 - 1075)	0.12 (-510 – 713)
Δ Androstenedione (ng/mL)			
0-24	0.03 (-4.6 – 2.6)	0.18 (-5.3 – 2.3)	-0.01 (-4.9 – 3.0)[#]
24-48	0.01 (-0.41 – 4.6)	0.21 (-3.5 – 2.6)	0.03 (-2.7 – 4.6)
48-72	0 (-0.57 – 0.315)	0.05 (-1.0 – 5.6)[b]	0 (-1.9 – 1.6)
Δ Testosterone (ng/dL)			
0-24	0 (-7.4 – 17.7)	0 (-22.2 – 7.2)	0 (-26.1 – 46.6)
24-48	0 (0 – 6.6)	0 (-1.6 – 25.6)	0 (-32.6 – 12.7)[@]
48-72	0 (0 - 0)	0 (-6.2 – 20.6)	0 (-51.5 – 12.2)
Δ DHT (pg/mL)			
0-24	248 (-315 - 927)	0 (-1258 - 1268)	0 (-1468 - 1082)[#]
24-48	119 (-280 - 854)	108 (-285 - 890)	4.4 (-553 - 971)
48-72	67.5 (-214 - 247)[a]	35.0 (-1041 - 1082)	1.4 (-103 - 492)

Delta values > 0 indicate that androgen concentrations decreased over time; values ≤ 0 indicate no change or increase in androgen concentrations over time. # indicates significant difference from healthy foals; @ indicates significance from SNS foals; “a” indicates significant difference from Δ0-24 within group. Symbols and letters of significance denote $P < .05$.

Abbreviations: DHEA; dehydroepiandrosterone; DHT, dihydrotestosterone; SNS, sick non-septic.

SUPPLEMENTARY TABLE 4: Androgen delta values in surviving and non-surviving foals. Data expressed as medians and ranges.

Δ DHEA (ng/mL)	**Survivor (n = 109)**	**Non-survivor (n = 36)**
0-24	0.00 (-60.4 – 40.0)	-7.20 (-43.7 – 23.5)[%]
24-48	0.40 (-5.70 – 34.8)	0.17 (-29.5 – 17.5)
48-72	0.02 (-0.71 – 44.8)	0.00 (-21.2 – 29.7)[a]
Δ Androstenedione (ng/mL)		
0-24	0.18 (-5.33 – 2.35)	-0.62 (-4.94 – 3.0)[%]
24-48	0.13 (-3.46 – 4.63)	-0.04 (-2.67 – 1.63)[%]
48-72	0.03 (-1.93 – 5.57)[a]	0.00 (-1.02 – 1.41)
Δ Testosterone (ng/dL)		
0-24	0 (-22.2 – 14.0)	-5.14 (-26.1 – 46.6)[%]
24-48	0 (-32.6 – 24.6)	-2.45 (-29.38 – 12.7)[%]
48-72	0 (-6.2 – 20.6)	0 (-51.5 – 6.3)
Δ DHT (pg/mL)		
0-24	0 (-1261 – 1268)	-604 (-1468 – 59.8)[%]
24-48	45.7 (-553 - 890)	0 (-323 – 971)
48-72	-17.7 (-1041 – 1372)	0 (-94.7 – 291)

Delta values > 0 indicate that androgen concentrations decreased over time; values ≤ 0 indicate no change or increase in androgen concentrations over time. % indicates significant difference from surviving foals; "a" indicates significant difference from Δ0-24 within group. Symbols and letters of significance denote P < .05.

Abbreviations: DHEA; dehydroepiandrosterone; DHT, dihydrotestosterone.

SUPPLEMENTARY TABLE 5: ACTH:androgen ratios in healthy and hospitalized foals over time. Data expressed as median and ranges.

	**Healthy (n = 80)**	**SNS (n = 64)**	**Septic (n = 81)**	**Sick non-NMS (n = 63)**	**NMS (n = 82)**
ACTH:DHEA					
0	0.24 (0.02-1.91)	1.52 (0.017-70.5)#	1.71 (0.02-33.1)#	2.3 (0.0017-58.0)#	0.91 (0.02-70.5)#
24	0.378 (0.01-8.02)	1.03 (0.020-25.9)	0.44 (0.01-12.0)	1.40 (0.012-25.9)	0.17 (0.01-11.8)
48	1.32 (0.16-4.33)[a]	3.39 (0.020-9.34)	1.20 (0.01-9.04)	2.23 (0.08-9.04)	0.51 (0.01-9.34)
ACTH:androstenedione					
0	20.57 (2.60-1013)	205 (3.14-4763)	219 (3.02-2301)	192 (3.14-4763)	210 (3.02-2302)
24	42.2 (3.17-1407)	71.0 (2.85-1273)[a]	44.7 (4.58-1582)	85.7 (3.60-1587)	18.1 (2.85-421)[a]
48	439 (29.8-1430)[a]	219 (3.31-906)	103 (4.88-1431)	333 (15.8-1163)	55.7 (3.31-810)#
ACTH:testosterone					
0	2.27 (1.02-10.5)	6.86 (0.81-149)	34.5 (0.70-156)#	6.21 (0.70-132)	21.6 (2.11-155.8)#
24	3.44 (0.43-7.38)	6.72 (0.81-186)	3.54 (0.50-64.4)	7.64 (0.81-186.6)	3.54 (0.50-46.7)[a]
48	6.98 (3.32-11.4)	4.62 (1.05-35.4)	2.58 (0.43-238)	4.0 (1.35-35.4)	4.16 (0.43-39.6)[a]
ACTH:DHT					
0	2.27 (1.02-10.5)	6.86 (0.81-149)	34.5 (0.70-156)#	6.21 (0.70-132)	21.6 (2.11-155.8)#
24	3.44 (0.43-7.38)	6.72 (0.81-186)	3.54 (0.50-64.4)	7.64 (0.81-186.6)[a]	3.54 (0.50-46.7)
48	6.98 (3.32-11.4)	4.62 (1.05-35.4)	2.58 (0.43-238)	4.0 (1.35-35.4)	4.16 (0.43-39.6)

# indicates significant difference from healthy foals; @ indicates significance from SNS foals; $ indicates significant difference from sick non-NMS foals; “a” indicates significant difference from time zero within group. Symbols and letters of significance denote P < .05.

Abbreviations: ACTH, adrenocorticotropic hormone; DHEA; dehydroepiandrosterone; DHT, dihydrotestosterone; SNS, sick non-septic; NMS, neonatal maladjustment syndrome.
